# The tumor immune microenvironment in primary cutaneous melanoma

**DOI:** 10.1007/s00403-024-03758-8

**Published:** 2025-01-18

**Authors:** Catherine Zilberg, Angela L. Ferguson, J. Guy Lyons, Ruta Gupta, Diona L. Damian

**Affiliations:** 1 The University of Sydney, NSW Camperdown, 2050 Australia; 2 Department of Tissue Pathology and Diagnostic Oncology, NSW Health Pathology, Royal Prince Alfred Hospital, Missenden Rd, Camperdown, NSW 2050 Australia; 3 Centenary Institute, The University of Sydney, Missenden Rd, Camperdown, NSW 2050 Australia; 4 Department of Dermatology, The University of Sydney at Royal Prince Alfred Hospital, Missenden Rd, NSW Camperdown, 2050 Australia; 5 Melanoma Institute Australia, 40 Rocklands Rd, NSW Wollstonecraft, 2065 Australia

**Keywords:** Skin neoplasms, Melanoma, Immunology, Tumor microenvironment

## Abstract

Melanoma is an immunogenic tumor. The melanoma tumor immune microenvironment (TIME) is made up of a heterogenous mix of both immune and non-immune cells as well as a multitude of signaling molecules. The interactions between tumor cells, immune cells and signaling molecules affect tumor progression and therapeutic responses. Understanding the composition and function of the TIME in primary cutaneous melanoma is useful for prognostication and therapeutic decisions. This review provides an overview of the components of the TIME in primary cutaneous melanoma, and their influence on clinical outcomes.

## Tumor infiltrating lymphocytes

Tumor Infiltrating lymphocytes (TILS) are lymphocytes that directly oppose or surround tumor cells [1]. TILS are composed of heterogenous cell types, and there are other immune cells associated with tumors in addition to TILS. Clemente et al. assessed 285 consecutive cases of stage I-II primary cutaneous melanoma. Greater TIL infiltration had prognostic significance for overall survival (OS) at both 5 and 10 years after diagnosis [2]. In a recent meta-analysis, including melanomas of all stages, TILS were correlated with a favorable prognosis for OS, recurrence free survival (RFS) and disease specific survival (DSS) [3]. Kumpers et al. investigated the immune cell infiltrate of primary cutaneous melanoma from patients who later received immune checkpoint inhibitor therapy for widespread disease.

Increased tumor immune infiltration in primary melanoma prior to treatment was associated with significantly improved OS (60% versus 11% 5 year survival) for patients with brisk and non-brisk lymphocytic tumor infiltrates respectively, and a nonsignificant trend for increased PFS [4].

### T cells

There are many subtypes of T cells that infiltrate tumors (Table 1). Studies are constrained by limited and non-specific markers that are used for the definition of cell populations. Higher proportion and number of T cells infiltrating a tumor is associated with an anti-tumor immune response [5]. High-throughput sequencing of the T cell receptor β-chain, and calculation of the T cell fraction (i.e. the fraction of T cells from all nucleated cells) from 199 primary cutaneous melanomas demonstrated higher proportion of T cells was associated with increased PFS [5]. Naïve T cells have not yet encountered antigen and are undifferentiated in terms of their functionality. Naïve T cells, which are poorly characterized in primary cutaneous melanoma, are unable to orchestrate an effective anti-tumor response [6]. Vermi et al. identified high levels of naïve T cells (CD45RA^+^, CD27^+^) in primary cutaneous melanoma [6].

**Table 1:** Summary of T cell subtypes and their immune function

Cell	Phenotype	Function
Cytotoxic T cells	CD8^+^ T cells (GZB^+^)	Recognize and specifically kill pathogens and tumor cells through death receptor binding and release of cytotoxic granules.
Naïve T cells	CD4^+^ or CD8^+^ cells (CD45RA^+^, CD27^+^)	T cells that have not encountered antigen. After antigen encounter, they proliferate and differentiate into effector and memory T cells.
CD4^+^ T helper cells	CD4^+^ T cells	Various function depending on subtype. Activate various immune cells and orchestrate immune responses.
CD4^+^ T regulatory cells	CD4^+^ FOXP3^+^T cells	Suppress activation and proliferation of immune cells.
Exhausted T cells	CD4^+^ or CD8^+^ T cells expressing inhibitory receptors– PD1, LAG3, TIGIT.	T cells with reduced effector functions and persistent expression of inhibitory receptors.
Precursors of exhausted T cells	CD8^+^TCF1^+^ PD1^+^ T cells	Associated with increased effector T cell responses following immune checkpoint blockade targeting PD1.

Cytotoxic T cells (CTLs) are effector CD8^+^ T cells that are equipped to kill tumor cells through the release of cytotoxic granules and/or death receptor binding.

Gartrell et al. demonstrated that higher stromal CD8^+^ CD3^+^ T cells are significantly correlated with increased disease specific survival (DSS) [7]. Piras et al. performed immunohistochemical (IHC) analysis of primary cutaneous melanoma specimens, and demonstrated that CD8^+^ lymphocytes at the base of the tumor mass exhibited a statistically positive, significant correlation with survival [8]. Activated CTLs, i.e. positive for granzyme B (GZB) were associated with significantly improved PFS and OS in stage II melanoma [9].

Using Opal multiplex immunohistochemistry in 66 stage II primary cutaneous melanomas, Attrill et al. showed that a higher proportion of a specific subset of CD8^+^ T cells, CD39^+^ CD103^+^ PD-1^−^ CD8^+^ T cells are seen in patients with a RFS greater or equal to 5 years [10]. These cells were increased in both the intra-tumoral and stromal regions of tumors with greater RFS.

Closer proximity between CD8^+^ T cells, tumor cells and HLADR^+^ macrophages was predictive of non-metastatic recurrence [11]. However, other studies have shown no predictive or prognostic effect of CD8^+^ T cells in primary melanoma [12, 13].

CD4^+^ T helper cells have many functions that depend on their subset– they coordinate immune responses, by both stimulating other immune cells and suppressing immune responses. Few studies focus on the role of CD4^+^ T cells in primary cutaneous melanoma. In one IHC study, greater infiltrates of CD4^+^ T cells and the IL-2 receptor (an early activation marker of T cells) were noted in regressing melanoma compared to non-regressing melanoma [14]. In another study, CD4^+^ T cells in primary biopsies of stage II melanoma were associated with favorable clinical outcomes [9]. CD4^+^ T cells appear to be less numerous than CD8^+^ T cells in the melanoma TIME [8].

CD4^+^ T FOXP3^+^ T regulatory cells are present in the primary melanoma TIME. CD4^+^ FOXP3^+^ T regulatory cells may make up a higher proportion of the CD4^+^ T cell compartment in melanoma compared to normal skin [15]. Increased gene transcripts associated with regulatory T cells were found in melanoma compared to nevi and normal skin [16]. In this meta-analysis by Shang et al. higher FOXP3^+^ T reg cell infiltration was associated with shorter OS in many cancers including melanoma. This was evaluated using IHC [17]. Miracco et al. investigated 66 vertical growth phase primary melanomas. Higher proportion of T regulatory cells in the tumor and peri-tumoral regions were significantly associated with recurrence [18].

Exhausted T cells demonstrate decreased activation and reduced cytokine production and cytolytic activity, leading to failure of cancer elimination [19]. These cells have increased expression of inhibitory receptors such as PD-1, CTLA-4, TIM-3, LAG-3 and TIGIT (among others). Greater gene transcripts associated with exhausted T cells were found in melanoma compared to nevi and normal skin [16].

Progenitors (or precursors) of exhausted T cells (TPEX) are a unique T cell subtype defined by higher levels of the transcription factor T-cell factor 1 (Tcf1) [20]. Siddiqui et al. used multiplex staining combined with digital image-based cell quantification and identified TCF1^+^ PD1^+^ CD8^+^ T cells preferentially localized to the stroma in stage I-III melanoma [20]. Miller et al. investigated TPEX cells in patients with advanced melanoma using quantitative immunofluorescence of pre-treatment biopsies of 25 patients with advanced melanoma who were treated with nivolumab and ipilimumab. The percentage of progenitor exhausted CD8^+^ T cells was calculated. Amongst responders, a higher proportion of progenitor exhausted CD8^+^ T cells was significantly associated with longer PFS and OS [21]. In a study by Sade-Feldman et al., more progenitor exhausted T cells were identified in tumors (baseline and post-treatment samples) from responders to anti-PD-1 treatment compared to non-responders [22].

There is significant scope for increasing our understanding the role of TPEX cells in primary cutaneous melanoma.

### B cells

The presence of B cells in melanoma is associated with a more favorable clinical outcome [10, 23]. Garg et al. used IHC for CD20 in tumor tissue, and single-cell mRNA expression of CD19/CD20 in primary cutaneous melanomas of > 1 mm Breslow thickness to demonstrate that higher CD20 staining and higher CD19/20 mRNA was associated with primary tumors that did not metastasize [23]. Primary melanomas with increased CD39^+^ CD103^+^ CD8^+^ PD1^−^ T cells demonstrated more B cells within the tumor and at the tumor-stroma interface, both of which were associated with greater RFS [10]. Higher B cell density together with higher density of CD25^+^ OX40^+^ T cells were associated with favorable prognosis [24].

B-cells may organize into follicle-like structures in melanoma (akin to tertiary lymphoid structures) [24, 25]. 72% of cases of primary cutaneous desmoplastic melanoma contained features of tertiary lymphoid structures which included B cells, CD8^+^ T cells and CD83^+^ dendritic cells (in T cell zones) [25]. In another study the authors used IHC to characterize ectopic lymphoid structures in 147 cases of primary cutaneous melanoma [26]. One quarter demonstrated aggregates of organized B cells which associated with CD45RO^+^ T cells, CD21^+^ cells (follicular dendritic cells) and the high endothelial venule marker, MECA-79.

Plasma cells develop from B cells and secrete immunoglobulin [27]. They express the pan-B cell marker CD19/20, as well as CD138 and CD38. Melanomas with clusters or sheets of plasma cells had significantly worse outcomes than melanomas with lower or no plasma cells although this occurrence was rare and mainly occurred in older patients [28].

## Antigen presenting cells

### Dendritic cells

Different types of dendritic cells (DCs) are present in the melanoma TIME with various effects. Salio et al. investigated the functionality (in vitro) of plasmacytoid DCs [29]. These cells can expand antigen-specific CD8^+^ T cells, which subsequently secrete type I IFN. There is a progressive accumulation of plasmacytoid DCs in invasive melanomas compared to MIS with no such cells in normal skin or benign nevi.

Ma et al. examined primary cutaneous melanoma tumors and positive and negative sentinel lymph nodes [30]. Higher CD11c staining of dendritic cells and presence of regression in primary tumors were protective against positive sentinel lymph node (LN) metastasis. Koelblinger et al. used IHC to evaluate expression of CD68, CD163 and CD11c (markers found on dendritic cells, macrophages and other cell types) in 112 primary cutaneous melanomas [31]. Expression was not associated with clinicopathologic outcomes [31].

Seidel et al. used immunofluorescence to investigate Langerhans cells (LC) (defined by expression of langerin and CD1a) in primary cutaneous melanoma, benign nevi and normal skin [15]. Tumor infiltrating LCs were less likely to express the activation marker CD40 compared to LCs in normal skin or benign nevi. Melanoma LCs often expressed PDL1 and had reduced expression of the co-stimulatory receptor CD80. Ladanyi et al. show a reduction in CD1a^+^ DCs in the epidermis overlying the vertical growth phase areas of SSMs and in the epidermis overlying nodular melanomas of the skin [32].

Dermal DCs, identified by expression of one (or multiple) of CD1a, DC-SIGN or CD206 were significantly increased in melanoma compared to benign nevi and normal skin [6]. Dermal DCs were predominantly located in the peritumoral region, often adjacent to lymphoid aggregates, with limited numbers of intratumoral DCs noted [6]. The DC maturation marker DC-SIGN was expressed on one third of dermal DCs from primary melanomas– implying a predominantly immature population of DCs, that may have reduced ability to stimulate effector CD8^+^ T cells [6]. In another IHC study of primary cutaneous melanoma, high peri-tumoral density of DC-LAMP^+^ cells, was associated with significantly longer OS at 5 years [32]. Similar results were found by Jensen et al., dense DC-LAMP^+^ DC infiltration in the peritumoural area was associated with better relapse free survival and melanoma specific survival by univariate analysis [13].

IHC staining for SOCS3, a marker of tolerogenic DCs, demonstrated increased expression (in a DC staining pattern) in melanoma compared to normal skin [16]. Double immunofluorescence of SOCS3 and CD11c confirmed localization of SOCS3 to dendritic cells. The same study demonstrated increased expression of additional gene transcripts associated with tolerogenic DCs.

### Macrophages

Tumor associated macrophages (TAMs) have an overall tumor promoting role in primary cutaneous melanoma. In a study by Salmi et al. numbers of CD68^+^ (non-specific marker) cells were significantly greater in malignant melanocytic lesions compared to benign nevi [33]. Higher CD68^+^ staining in tumor cell nests was associated with both locoregional and distant recurrence [33]. Similarly, melanomas with dense CD163^+^ cell infiltration in the tumor stroma, and CD68^+^ infiltration at the tumor front were associated with poorer OS [34]. In another IHC study, high macrophage counts (also using IHC staining of CD68) was associated with ulceration, Breslow thickness, mitotic rate, lymphovascular invasion and micro vessel density [35].

Bianchini et al. investigated 27 cases of melanoma and pigmented nevi [36]. These included 4 melanoma in situ (MIS) and 11 invasive primary cutaneous melanomas. MIS and invasive primary cutaneous melanoma had higher COX2^+^ CD68^+^ cells compared to metastatic lesions and benign nevi. These cells were preferentially located at the leading edge of the tumor and may play a role in disease progression.

The chemokine receptor CCR6, and its ligand CCL20 have been described to play a role in tumor progression in several cancers [37]. CCR6 is expressed in melanoma cells, in vivo addition of CCL20 leads to tumor growth and progression [38]. Within primary cutaneous melanoma specimens CCR6 is localized to the tumor area and CCL20 to the stroma. CCL20 was expressed in M1, M2 and M1/M2 like macrophages– identified using fluorescence confocal microscopy. Macrophages expressing CCL20, VEGF-A and TNF were associated with tumors that exhibited metastatic disease [38].

Adrenomedullin (ADM) is a vasodilator, but has additional functions in many cancers including angiogenesis and cell proliferation [39]. ADM has been detected in melanoma, where it is expressed on many cell types, including in immune cells such as macrophages and T cells [40]. Co-culture of a mouse leukemic macrophage cell line with melanoma cells resulted in increased ADM secretion from the macrophages [40]. ADM secreting macrophages have been shown to increase endothelial cell migration and tubule formation and increase tumor growth.

Some studies have not demonstrated an association between TAMs and poorer clinicopathologic outcomes [41]. CD68 and CD163 IHC staining was not associated with prognosis in 57 cutaneous melanoma cases [41]. Another study investigating 123 cutaneous melanomas identified M1, M2 and M1/2 type TAMs within tumors using multicolor fluorescence confocal microscopy [42]. CD163^+^CD11c^+^CD209^−^ cells were classified as M1-type, CD163^+^CD11c^−^CD209^+^ cells were classified as M2 type, and CD163^+^CD11c^+^CD209^+^ cells as mixed M1/2 type. The authors found no significant association of TAM density, size, and location, nor CD11c/CD209 subsets with DFS or OS. Multicolor fluorescence staining revealed CCL20, VEGF and TNF in CD68^+^ cells are present in higher levels in tumors that demonstrated progression [42].

### **NK cell**

There is limited research focusing on the roles of natural killer (NK) cells in the primary melanoma TIME. CD16, a non-specific marker of NK cells, was identified in 10 of 16 melanomas and 0 of 7 benign melanocytic lesions [43]. Intratumoral NK cell density did not affect prognosis in a study using opal multiplex immunohistochemistry and quantitative pathology-based immune-profiling [10].

### **Mast cells**

Mixed results regarding mast cells in the melanoma TIME have been reported.

Siiskonen et al., conducted an IHC study investigating the expression of tryptase and chymase, two major proteins in mast cell secretory granules [44]. Mast cell-specific tryptase and chymase positive cells was reduced in more invasive melanoma, lower scores were associated with reduced survival. Rajabi et al. analyzed tryptase using IHC, and TILS density, in primary cutaneous melanoma (stage I-III) [45]. Fewer intratumoral and peritumoral mast cells and TILS were found in higher stages of tumor depth in malignant melanoma. Contrary results were noted by Toth-Jakatics et al. [46]. IHC staining of anti-tryptase and VEGF to identify mast cells and VEGF^+^ mast cells in primary cutaneous melanoma and intradermal nevi demonstrated that mast cell density (and VEGF^+^ mast cell density) was significantly higher in melanoma compared to benign nevi [46]. Similar results were obtained by Ribatti et al. [47].

### **Neutrophils**

Within the cancer TIME neutrophils cells can be classified into two sub-types N1 and N2 [48]. N1 neutrophils are thought of as having anti-tumor functions, whereas N2 are considered to be pro-tumorigenic [48].

Jensen et al. used IHC to investigate the prognostic role of neutrophils in 186 primary cutaneous melanomas (stage I/II) [13]. CD66b cells (designated neutrophils) were associated with poorer prognosis. Melanoma cancer stem cells in vitro could alter the phenotype of neutrophil-like cells to a pro-tumorigenic N2 phenotype and increased activation of N2 associated pathways [49]. This included phosphorylation of STAT3 and ERK1/2, overexpression of CXCR2/NFκβ and P38 activation. Cells treated with conditioned medium from melanoma stem cells had increased ROS production, neutrophil extracellular trap release and greater secretion of matrix metalloproteinase 9 and the pro-tumorigenic cytokines IL-6 and IL-8. Melanoma cells may be able to promote neutrophil switch to a more immunosuppressive, pro-tumorigenic phenotype [49].

### **Cytokines and chemokines**

Melanoma demonstrates a TIME with increased levels of suppressive cytokines and growth factors, and reduced levels of pro-inflammatory, cytotoxic cytokines [16, 50, 51]. TGFβ is an immunosuppressive cytokine secreted by tumor cells and components of the microenvironment in melanoma [52–54]. TGFβ exists in 3 isoforms– TGFβ1, TGFβ2 and TGFβ3. mRNA profiling of TGFβ2 in 124 melanocytic lesions including nevi, MIS, primary and metastatic melanoma demonstrated a progressive decline in TGFβ2 expression from metastatic lesions, to deeper primary lesions, to superficial lesions to nevi [52]. Another study used IHC to determine expression patterns of TGFβ2 and AMBRA1 in primary cutaneous melanoma [53]. Increased tumoral TGFβ2 was significantly associated with loss of AMBRA1 (roles in autophagy and cell cycle regulation), ulceration and metastasis. Van Belle et al. used IHC staining of all 3 TGFβ isoforms and demonstrated that there is a progressive increase in each from melanocytic lesions, to thicker lesions to metastatic lesions [54]. TGFβ may play a role in tumor progression.

The T_h_1 cytokine IFNγ is the primary effector cytokine of the CTL response. There is reduced IFNγ (as detected by quantitative RT-PCR) in melanoma compared to dysplastic nevi [16]. There are greater levels of mitogenic cytokines and growth factors in melanoma compared to benign melanocytic nevi, including CXCL1, CXCL2, IL-6, IL-8, oncostatin M (OSM), nerve growth factor (NGF), granulocyte-macrophage colony-stimulating factor (GM-CSF), VEGF-A and platelet derived growth factor β (PDGF-subunit β) [16]. Regressing primary cutaneous melanoma demonstrates greater mRNA levels of CD35 (complement receptor 1), lymphotoxin-α and IL-2 compared to non-regressing melanoma [50].

The suppressive cytokine IL-10 may be elevated in melanoma. Itakura et al. found increased mRNA and protein expression of IL-10 in invasive melanoma compared to MIS and melanocytic nevi [51]. In primary melanomas, tumor cell IL-10 mRNA content is associated with increasing Clark and Breslow thickness.

CXCR4 is a chemokine receptor which binds to CXCL12 [55]. Meta analysis of 13 studies investigating overall CXCR4 expression in melanoma tissue (calculated by IHC staining or qPCR) in primary and metastatic tissue demonstrated that over expression of CXCR4 is associated with ulceration, increased thickness and lymph node spread [55].

The cytokine and chemokine milieu in primary cutaneous melanoma is one characterized by increased mitogenic cytokines, reduced IFN-γ and increased IL-10 levels, thereby facilitating tumor survival and progression.

## IDO

IDO, an immunosuppressive enzyme, has been demonstrated to be present in primary cutaneous melanoma [56, 57]. In a cohort of 99 primary melanomas from immunocompetent patients, higher expression of IDO was associated with higher Breslow thickness, ulceration and mitotic rate [57]. Independent of Breslow thickness and melanoma stage, greater IDO staining (both on tumor cells and other cells of the TME) was associated with reduced PFS [57].

## Immune checkpoint receptors and ligands

### PD-1 and PD-L1

Programmed Cell Death Protein 1 (PD-1) is typically expressed by activated T cells, B cells, NK cells, macrophages, dendritic cells and some tumor cells. Programmed Cell Death Ligand 1 (PD-L1) can be expressed by T and B cells, macrophages, dendritic cells, tumor cells, epithelial cells, endothelial cells. Interactions between PD-1 and its ligand PD-L1 leads to inhibition of the adaptive and innate arms of the immune system and is a well-described mechanism behind tumor evasion, and suppression of the anti-cancer immune response.

Kaunitz et al. evaluated melanoma on chronic sun damaged skin where more than half the cases demonstrated high staining for PD-L1 [58]. High PD-L1 expression was significantly correlated with moderate to high CD8^+^ T cells. However, another study did not find correlation with PD-L1 expression and either CD3 or CD8 expression [59]. PD-L1 staining was associated with thicker tumors and nodular melanoma subtype. PD-L1 expression in primary tumors may be discordant with PD-L1 expression in metastatic lesions [60]. In a cohort of 17 primary melanoma tumors and 18 cutaneous metastases the levels of PD-1 on CD4^+^ and CD8^+^ TILS were investigated using flow cytometry [61]. A gradual increase in expression of PD1 on CD4^+^ or CD8^+^ CD3^+^ T cells in thicker and more advanced melanomas was noted. Upregulation of PD-1 on CD8^+^ TILs was also higher in thicker primary tumors with Breslow ≥ 1 mm compared with Breslow < 1 mm [61]. In a meta-analysis by Xu et al., positive PD-L1 expression was significantly correlated with OS in metastatic melanoma [62]. Yang et al. conducted a meta-analysis including 1062 patients from 13 studies [63]. Overexpression of PD-L1 was not associated with OS or PFS. However, PD-L1 overexpression was correlated with absence of lymph node metastases.

### LAG-3 and TIGIT

Lymphocyte Activation Gene 3 (LAG-3) is a checkpoint molecule that can be expressed on NK cells, B cells, TILS and DCs amongst others. It binds to MHC II and inhibits T cell proliferation and activation. T cell immunoreceptor with immunoglobulin and ITIM domain (TIGIT) is expressed by NK cells, T regulatory cells and CD8^+^ T cells and results in increased suppressive cytokines.

Lee et al. investigated the expression of TIGIT, LAG-3 and PD-1 in 124 cases of cutaneous melanoma by IHC [64]. LAG-3 expression was significantly correlated with poor clinicopathologic variables including– deeper Breslow thickness, vertical growth, mitoses, lymph node involvement and advanced AJCC stage [64]. Similar associations were noted for higher TIGIT expression [64]. LAG-3 expression and TIGIT expression were both associated with worse OS and RFS [64].

Naimy et al. used Nanostring technology to evaluate the differential expression of genes by RNA analysis in primary cutaneous melanoma with brisk TIL infiltration compared to tumors with absent TIL infiltration [65]. Both LAG-3 and TIGIT expression was increased in the cohort with the brisk TIL infiltrate [65]. Using multiplex immunofluorescence, LAG-3 was highly expressed on CD8^+^ T cells and to a lesser extent on T regulatory cells, CD4^+^ T cells and NK/NKT cells [65]. TIGIT was highly expressed in T regulatory cells, CD8^+^ T cells and on a minority of CD4^+^ T cells [65]. LAG-3 and TIGIT were often co-expressed with PD-1 [65].

## Cancer associated fibroblasts

Cancer-Associated Fibroblasts (CAFs) are cells within the melanoma microenvironment that differ from normal fibroblasts. Activated by cancer cells, CAFs support tumor growth, invasion, immune evasion, angiogenesis, and treatment resistance [66]. Papaccio et al. studied mRNA and protein levels of tumor-associated markers in CAFs from melanoma specimens, compared to normal fibroblasts [67]. They found higher expression of matrix metalloproteinase 2, interleukin 8 (IL-8), and PD-L2 in CAFs. Protein analysis showed increased hepatocyte growth factor, stem cell factor, basic fibroblast growth factor, and vascular endothelial growth factor (VEGF).

CAF supernatant accelerated melanoma cell migration and reduced the cytotoxic effects of paclitaxel, suggesting CAFs promote tumor progression and treatment resistance [67]. Another study examined melanoma’s effect on fibroblasts, comparing control fibroblasts to CAFs exposed to melanoma cells or conditioned media [68]. CAFs showed greater migration, motility, lactate production, MAPK activation, and higher levels of pro-angiogenic factors and cytokines (e.g., CCL2, IL-8, VEGF) than controls, indicating a stronger influence on tumor growth [68]. Ersek et al. found that CAFs suppress CD8^+^ T cell activity by downregulating CD69, reducing granzyme B release, increasing immune checkpoint receptors TIGIT and BTLA, and increasing chemokine (C-X-C motif) ligand 2, which repels CD8^+^ T cells [69]. Ziani et al. showed that CAFs protect melanoma cells from NK cell-mediated lysis by secreting matrix metalloproteinases, which reduce the NKG2D-dependent cytotoxic activity of NK cells [70].

## Melanoma cells influence on the tumor microenvironment

Melanoma cells interact with immune and stromal cells in the TIME to promote tumor survival. They interfere with cytotoxic T cells, alter antigen-presenting cells, modify cytokine production, express immune checkpoint proteins, and evade immune-mediated cell death through expression and secretion of metabolites, proteins, circulating tumor RNAs or DNAs [68, 71–74]. For instance, melanoma cells increase expression of anti-apoptotic proteins like FLICE Inhibitory Protein (FLIP) and inhibitors of apoptosis (IAP) [72, 75].

Melanoma cells can also stimulate TIME components to become more tumorigenic– for example, Simiczyjew et al. demonstrated that melanoma cells alter the metabolism and phenotype of adipocytes, promoting tumor growth by increasing secretion of lactate and fatty acids [73]. Similarly, melanoma cells induce CAFs to increase motility, proteolytic activity, and angiogenesis, while keratinocytes become less differentiated and form connections with cancer cells, enhancing tumor progression [68, 74]. Bachmann et al. highlighted that melanoma cells modify adhesion molecule expression [76]. As outlined in a prior section, melanoma cells can express immune checkpoint proteins which can interact with immune cells bearing respective receptors and alter their activity.

## Summary

The immune microenvironment of primary cutaneous melanoma is complex, involving various cell populations and their interactions. Studies are limited by nonspecific markers for defining these cells. Whilst several studies have demonstrated an anti-tumor role for CD8^+^ T lymphocytes, some studies have provided contrasting results. CD4^+^ T regulatory cells may promote tumor survival. B cells have been linked to favorable outcomes. A subset of exhausted T cells, progenitors of exhausted T cells, have been associated with favorable treatment responses. Tumor-associated macrophages (TAMs) and cancer-associated fibroblasts (CAFs) tend to support tumor growth, immune evasion, and treatment resistance. CAFs also suppress CD8^+^ T cell activity and increase tumor cell motility. The roles of NK and mast cells remain largely unclear, while neutrophils can adopt tumor-promoting functions. Suppressive cytokines (TGFβ, IL-10) are elevated, and cytotoxic cytokines (IFNγ) are reduced. Most studies have reported that higher immune checkpoint receptor expression (i.e. LAG-3, PD-1, and TIGIT) is associated with poorer clinicopathologic variables such as greater Breslow thickness and mitoses. PD-L1 positivity has been associated with increased OS in metastatic melanoma.

## Data Availability

No datasets were generated or analysed during the current study.
